# Risk factors associated with abscess formation among patient with leg erysipelas (cellulitis) in sub-Saharan Africa: a multicenter study

**DOI:** 10.1186/s12895-015-0037-7

**Published:** 2015-12-15

**Authors:** Palokinam Vincent Pitché, Bayaki Saka, Ahy Boubacar Diatta, Ousmane Faye, Boh Fanta Diané, Abdoulaye Sangaré, Pascal Niamba, Christine Mandengue, Léon Kobengue, Assane Diop, Fatimata Ly, Mame Thierno Dieng, Alassane Dicko, Maciré Mohamed Soumah, Mohamed Cissé, Sarah Hamdan Kourouma, Isidore Kouassi, Taniratou Boukari, Sefako Akakpo, Dadja Essoya Landoh, Kissem Tchangaï-Walla

**Affiliations:** Service de dermato-vénéréologie, CHU Sylvanus Olympio, Université de Lomé, 08 BP 81056 Lomé 08, Togo; Service de dermatologie, CHU Le Dantec, Dakar, Université Cheik Anta Diop, Dakar, Sénégal; Service de dermatologie, CNAM, Bamako, Université de Bamako, Bamako, Mali; Service de dermatologie-MST, CHU Donka, Conakry, Université de Conakry, Conakry, Guinée; Centre de dermatologie, CHU Treichville, Université de Cocody, Cocody, Côte d’Ivoire; Service de dermatologie CHU Yaldago Ouédraogo, Ouagadougou, Université de Ouagadougou, Ouagadougou, Burkina Faso; Service de dermatologie, Clinique universitaire des Montagnes, Bangangté, Cameroun; Service de dermatologie, CHU Bangui, Université de Bangui, Bangui, Centrafrique; Division de l’Epidémiologie, Ministère de la santé du Togo, Lomé, Togo

**Keywords:** Erysipelas of leg, Lower limbs cellulitis, Abscess formation, Risk factors, Sub-Saharan Africa

## Abstract

**Background:**

Abscess formation is a frequent local complication of leg erysipelas. In this study we aimed at identifying factors associated with abscess formation of leg erysipelas in patients in sub-Saharan African countries.

**Method:**

This is a multicenter prospective study conducted in dermatology units in eight sub-Saharan African countries from October 2013 to September 2014. We performed univariate and multivariate analysis to compare characteristics among the group of patients with leg erysipelas complicated with abscess against those without this complication.

**Results:**

In this study, 562 cases of leg erysipelas were recruited in the eight sub-Saharan African countries. The mean age of patients was 43.67 years (SD =16.8) (Range: 15 to 88 years) with a sex-ratio (M/F) of 5/1. Out of the 562 cases, 63 patients (11.2 %) had abscess formation as a complication. In multivariate analysis showed that the main associated factors with this complication were: nicotine addiction (aOR = 3.7; 95 % CI = [1.3 – 10.7]) and delayed antibiotic treatment initiation (delay of 10 days or more) (aOR = 4.6; 95 % CI = [1.8 – 11.8]).

**Conclusion:**

Delayed antibiotics treatment and nicotine addiction are the main risk factors associated with abscess formation of leg erysipelas in these countries. However, chronic alcohol intake, which is currently found in Europe as a potential risk factor, was less frequent in our study.

## Background

Cellulitis is an infection of the deep layers of the skin (dermis and hypodermis), mainly caused by streptococcus species [1]. Its localization on the face has become rare meanwhile; the lower limbs localization is currently more frequent. There are various risk factors associated with lower extremities cellulitis such as; lymphoedema, site of entry, leg oedema, venous insufficiency, traumatic wound, leg ulcers, toe-web intertrigo, excoriated leg dermatosis [2, 3]. Various complications might occur during the course of cellulitis. The local and general complications frequently reported are abscess, superficial necrosis or deep venous thrombosis, these complications might occur as from the first days, but relapse of cellulitis and its sequelae occur sometimes later [4]. Abscess formation is the most frequent local complication [4, 5], furthermore, a study conducted in Europe showed that risk factors identified to be associated with abscess formation were chronic alcohol intake, as well as, delayed antibiotic treatment initiation [6]. In Africa, very few data on the risk factors for abscess formation in cellulitis’s patients have been published. In this multicenter study, we aim at identifying risk factors associated with abscess formation among patients presenting with cellulitis of lower extremities in sub-Saharan African countries.

## Method

### Type and population of the study

This is a multicenter prospective study conducted in dermatology units of eight sub-Saharan African countries. There were six countries from West Africa (Togo, Senegal, Mali, Côte d’Ivoire, Guinea, and Burkina Faso) and two central African countries (Central African Republic and Cameroon). We recruited patients more than 15 years old who attended dermatology consultation for the onset of leg’s cellulitis. The study period was twelve months from October, 2013 to September, 2014.

### Data collection

A validated questionnaire from two study centers was used to collect data. The variables collected from each patient were:

i) Sociodemographic and anamnesis’ data i.e. age, sex, use of non-steroid anti-inflammatory drugs and use of cataplasm before consultation. ii) Clinical data i.e. the site of cellulitis, the degree of pain, the occurrence of fever and/or shivering, cutaneous signs: phlyctenas, and purpura, satellite adenopathy, and skin complications such as abscess formation and necrosis.

During the consultation, patients were asked for the date of onset of cellulitis (debut of at least one of the four following signs and symptoms: pain, redness, swelling of the leg and warm leg) and the date of antibiotic initiation if antibiotic was started before consultation. In general most of patients have started antibiotics the day of the consultation, but few patients had already started antibiotics before the consultation. A patient was considered to have a delay in antibiotic initiation when the period between the onset of cellulitis and antibiotic initiation exceeded 10 days. Patients were followed up during hospitalization to record outcome information. During patient’s hospitalization, the onset of an abscess was diagnosed by a physician. Once diagnosed, the abscess was incised and drained.

Other variables were also collected from patients through history taking, as well as trough clinical and biological examinations, they were: iii) In medical history, we searched for chronic alcohol intake, sedentary life style and nicotine addiction: iv) In clinical examination, we looked for point of entry (traumatic wound, vascular ulcers, excoriations, intertrigo of intertie), pitting edema, varicous veins, arteriopathy, previous surgery of the leg, deep venous thrombosis, obesity (BMI ≥30), hypertension, neurologic disorders and/or use of bleaching products; v) in Lab test we performed: Glucose and HIV tests.

### Statistical analysis

Data were recorded using Epi Info software version 3.1 and analyzed in SPSS® 20.0 (IBM Corporation, Armonk NY, USA) software. For continuous variables, means and standard deviations were calculated while for categorical variables we calculated proportions. Our primary outcome of interest was patients who had complication of cellulitis with abscess formation compare to those without abscess formation. Chi square or Fisher exact test were used when appropriate in univariate analysis. Multivariate backwards stepwise logistic regression analysis was performed to identify independent risk factors for the dichotomous outcome complication of cellulitis with abscess formation or absence of abscess. All variables significant during univariate analysis at a *p*-value less than 0.2 were then included in the multivariate analysis to assess the adjusted effect and derive the adjusted odds ratio (aOR) of each on the primary outcome. We allocated the value “1” to the dependent dichotomous variable if cellulitis becomes complicated with abscess formation, and the value 0 otherwise. A 95 % level of confidence was applied throughout.

### Ethical issues

Ethics clearance was obtain from each Ethics Committee board of the Universities of the 8 countries participating in this study. The participant signed an informed consent form, after the verbal explanation was delivered by the investigating officers. The survey was anonymous and confidential.

## Results

From October, 2013 to September, 2014, a total of 562 cases of leg’s cellulitis were recruited in the eight participating countries. The mean age of patients was 43.7 years (SD = 16.8) ranging from15 to 88 years. The sex-ratio (M/F) was 5/1. Out of 562 cases, 63 (11.2 %) had abscess formation as complication. Chronic alcohol intake was found in 2/63 patients (3.2 %), while nicotine addiction was found in 7/63 patients (11.1 %). Meanwhile, 1/63 (1.6 %) patient was infected with HIV. Concerning the point of entry, 12/63 (19 %) had inter-toes intertrigo and 43/63 (68.3 %) had neglected wounds on the legs (Table 1). Delayed antibiotic treatment was found in 492/562 patients (87.5 %) (Table 1).

**Table 1:** Risk factors associated with abscess formation of leg erysipelas, univariate analysis

Characteristics	Total	Abscess formation		OR	95 % CI	*P*
	*N* = 562 (%)	Yes, *n* (%)	No, *n* (%)			
Age						
*<25 years*	*76 (13.5)*	*10 (13.2)*	*66 (86.8)*	1	-	0.43
*25-35 years*	*134 (23.9)*	*11 (8.2)*	*123 (91.8)*	0.59	[0.24 – 1.46]	
*>35 years*	*352 (62.6)*	*42 (11.9)*	*310 (88.1)*	0.89	[0.43 – 1.86]	
Gender						
* Male*	*223 (39.7)*	*31 (13.9)*	*192 (86.1)*	1.55	[0.92 – 2.62]	0.10
* Female*	*339 (60.3)*	*32 (9.4)*	*307 (90.4)*	1		
Obesity						
*Yes*	*230 (40.9)*	*23(10.0)*	*200 (90.0)*	0.81	[0.47 – 1.40]	0.45
*No*	*332 (59.1)*	*40(12.0)*	*292 (88.0)*	1		
Chronic alcohol intake						
*Yes*	*18 (3.2)*	*2 (11.1)*	*16 (88.9)*	0.99	[0.22 – 4.41]	0.67
*No*	*544 (96.8)*	*61 (11.2)*	*483 (88.8)*	1		
Diabetes						
*Yes*	*27 (4.8)*	*2 (7.4)*	*25 (92.6)*	0.62	[0.14 – 2.69]	0.76
*No*	*535 (95.2)*	*61 (11.4)*	*474 (88.6)*	1		
Hypertension						
*Yes*	*81 (14.4)*	*10 (12.3)*	*71 (87.7)*	1.14	[0.55 – 2.34]	0.73
*No*	*481 (85.6)*	*53 (11.0)*	*428 (89.0)*	1		
Sedentary						
*Yes*	*85 (15.1)*	*12 (14.1)*	*73 (85.9)*	1.37	[0.70 – 2.70]	0.36
*No*	*477 (84.9)*	*51 (10.7)*	*426 (89.3)*	1		
Nicotine addiction						
*Yes*	*29 (5.2)*	*7 (24.1)*	*22 (75.9)*	2.71	[1.11 – 6.63]	0.03
*No*	*533 (84.8)*	*56 (10.5)*	*477 (89.5)*	1		
HIV infection						
*Yes*	*16 (2.8)*	*1 (6.2)*	*15 (93.8)*	0.52	[0.07 – 4.01]	0.44
*No*	*546 (97.2)*	*62 (11.4)*	*484 (88.6)*	1		
Pitting oedema						
*Yes*	*130 (23.1)*	*20 (15.4)*	*110 (84.6)*	1.65	[0.93 – 2.91]	0.08
*No*	*432 (76.9)*	*43 (10.0)*	*389 (90.0)*	1		
Varicous vein						
*Yes*	*19 (3.4)*	*2 (10.5)*	*17 (89.5)*	0.93	[0.21 – 4.21]	0.64
*No*	*543 (96.6)*	*61 (11.2)*	*482 (88.8)*	1		
Obstructive arteriopathy						
*Yes*	*5 (0.9)*	*1 (20.0)*	*4 (80.0)*	1.99	[0.22 – 18.14]	0.45
*No*	*557 (99.1)*	*62 (11.1)*	*495 (88.9)*	1		
Use of bleaching agents						
*Yes*	*97 (17.3)*	*5 (5.2)*	*92 (94.8)*	0.38	[0.15 – 0.98]	0.04
*No*	*465 (82.3)*	*58 (12.5)*	*407 (87.5)*	1		
Previous history of phlebitis						
*Yes*	*2 (0.4)*	*0 (0.0)*	*2 (100.0)*	-	-	0.79
*No*	*560 (99.6)*	*63 (11.2)*	*497 (88.8)*			
Previous history of surgery of the leg						
*Yes*	*6 (1.1))*	*2 (33.3)*	*4 (66.7)*	4.06	[0.73 – 22.62]	0.14
*No*	*556 (98.9)*	*61 (11.0)*	*495 (89.0)*	1		
Neurologic disorders						
*Yes*	*1 (0.2))*	*0 (0.0)*	*1 (100.0)*	-	-	0.88
*No*	*561 (99.8)*	*63 (11.2)*	*498 (88.8)*			
Intertrigo of intertoe						
*Yes*	*161 (28.6)*	*12 (7.5)*	*149 (92.5)*	0.55	[0.29 – 1.07]	0.07
*No*	*401 (71.4)*	*51 (12.7)*	*350 (87.3)*	1		
Neglected wound on the leg						
*Yes*	*324 (57.7)*	*43 (13.3)*	*281 (86.7)*	1.67	[0.95 – 2.92]	0.07
*No*	*238 (42.3)*	*20 (8.4)*	*218 (91.6)*	1		
Delayed of antibiotics treatment at onset of erysipelas						
*<3 days*	*104 (18.5)*	*8 (7.7)*	*96 (92.3)*	1	-	<0.001
*3-10 days*	*295 (52.5)*	*23 (7.8)*	*272 (92.2)*	1.01	[0.44 – 2.33]	
*>10 days*	*93 (16.5)*	*28 (30.1)*	*65 (69.9)*	5.17	[2.22 – 12.05]	
Use of non steroidanti inflammatory drugs						
*Yes*	*207 (36.5)*	*35 (16.9)*	172 (83.1)	2.38	[1.40 – 4.04]	0.001
*No*	*355 (63.5)*	*28 (7.9)*	327 (92.1)	1		
Use of cataplasms and decoctions before consultation						
*Yes*	*104 (18.5)*	*21 (20.2)*	*83 (79.8)*	2.51	[1.41 – 4.45]	0.001
*No*	*458 (81.5)*	*42 (9.2)*	*416 (90.8)*	1		

In univariate analysis, associated factors of abscess formation in patient with cellulitis of the leg were: nicotine addiction (OR = 2.71; 95 % CI = [1.1 – 6.6]), the use of bleaching agents (OR = 0.4; 95 % CI = [0.2 – 0.9]), delayed antibiotics treatment initiation (delay of 10 days or more): OR = 5.2; 95 % CI = [2.2 – 12.1]); the use of non steroid anti-inflammatory drugs before consultation (OR = 2.4; 95 % CI = [1.4 – 4.1]); the use of cataplasms and decoctions before consultation (OR = 2.5; 95 % CI = [1.4 – 4.5]) (Table 1).

Furthermore, on multivariate analysis, associated risk factors with abscess formation that remained statistically significant were nicotine addiction (aOR = 3.7; 95 % CI = [1.3 - 10.7]) and delayed antibiotics treatment initiation (delay of 10 days or more: aOR = 4.6; 95 % CI = [1.8 - 11.8]) (Table 2).

**Table 2:** Risk factors associated with abscess formation of erysipelas of the leg, multivariate analysis

Characteristics	aOR	95 % CI for aOR
Nicotine addiction		
	3.75	[1.32 ; 10.70]
Delayed antibiotics treatment at onset of erysipelas		
*More than 10 days*	4.65	[1.84 ; 11.80]

## Discussion

This was a multicenter prospective study carried out in dermatology units in eight sub-Saharan African countries, aiming at identifying risk factors associated with abscess formation as complication of lower extremities cellulitis. Nicotine addiction and delayed in antibiotic treatment initiation were identified as the risk factors associated with abscess formation of leg cellulitis in these countries. Meanwhile, biological analysis of this skin infection was not performed.

Abscess formation is the most frequent complication of cellulitis. In our study we found 11.2 % of this complication. A meta-analysis of this skin disease conducted within a period of 20 years showed that abscess formation, necrosis and/or deep venous thrombosis complicated 3 % to 12 % of lower limb cellulitis [4]. Krasagakis et al [5] found (46/145) 31.7 % of cases of leg cellulitis to be complicated with abscess formation, while others authors, Picard et al. [6], Mahe et al. [7], Crickx et al. [8] observed 7.9 %, 9.9 % and 3.6 % of cases, respectively. Meanwhile, a monocenter study conducted in Lomé (Togo) detected only (3/67) 4.5 % cases of this complication [9].

Abscess formation is the most frequent, and the main cause of morbidity in lower extremities cellulitis, which prolonged patient’s hospitalization and increased the financial coast for both patient and the community.

In our study we identified two risk factors associated with abscess formation of leg cellulitis: delayed antibiotics treatment initiation and nicotine addiction. Delayed antibiotics treatment initiation increases the risk of abscess formation by 1.4 to 4.6 as reported in many other studies [5, 6, 8, 9], thereby, bacteria would become more pathogenic, and invade the deep layers of the skin. We could not give pathophysiological relation between this complication and nicotine addiction. Nevertheless, nicotine addiction could induced an immune depression as in chronic alcoholism, which was not currently found in our study compared to another publication [6].

Furthermore, obesity, diabetes, HIV infection and the use of bleaching products, which are more frequent in sub-Saharan Africa [5, 10], were not found as risk factors associated with local complications and severe cellulitis in this study.

### Limitations

This study did not investigate biological aspects of skin infection, which could explain the pathophysiological aspects of abscess formation among patients with leg cellulitis. Also, socioeconomic condition of patients may have influenced the delay in antibiotic initiation. Finally, some recall bias could have occurred during recording of anamnesis information. However, in the eight participating dermatology units, investigation officers have used a validated structured data collection tool, in order to, reduce information bias across countries.

## Conclusion

In this study we found that abscess formation is a very frequent complication of leg cellulitis, which is mainly due to nicotine addiction and delayed antibiotics treatment initiation. Knowing these risk factors may help early detection and treatment of this complication.
